# Trustworthy AI for radar vital signs: detecting and mitigating gender bias in healthcare

**DOI:** 10.1007/s40747-026-02296-2

**Published:** 2026-04-25

**Authors:** Nour Ghadban, Jonathan Cooper, Julien Le Kernec

**Affiliations:** James Watt School of Engineering, University of Glasgow, Glasgow, Scotland UK

**Keywords:** Healthcare artificial intelligence, Radar-based vital sign monitoring, Algorithmic gender bias, Bias detection and mitigation, Algorithmic fairness, Trustworthy AI

## Abstract

Artificial intelligence (AI) has emerged as a fundamental component in modern healthcare, particularly in noninvasive monitoring of vital signs using radar-based systems. However, algorithmic fairness concerns, such as gender bias, can undermine trust in these systems. This study investigates the impact of gender representation in training data on the accuracy and fairness of radar-based vital sign estimation. We trained machine-learning models on 60 dataset configurations–male-only, female-only, balanced, male-dominant and female-dominant–each containing 640 radar-derived samples (3200 total). Models trained on female-dominant data achieved the highest classification accuracy (94.5%) and lowest regression error (RMSE = 0.70), whereas male-only datasets performed worst (accuracy = 78.2%, RMSE = 1.80). Disparate impact analysis revealed up to a 16.5% performance advantage for female-skewed training data, and multiple fairness metrics, including disparate impact ratio and statistical parity difference, were employed to quantify bias across subgroups. To address these disparities, we implemented a multilevel mitigation framework integrating TimeGAN-based data augmentation, fairness-aware learning constraints, and threshold adjustment. This approach reduced the bias score from 0.0261 to 0.00005 ($\textit{p} < 0.001$) while maintaining a high F1 score ($> 0.98$). Our results underscore the necessity of balanced and diverse datasets and demonstrate that incorporating fairness-aware strategies can yield equitable and trustworthy AI systems for clinical vital sign monitoring.

## Introduction

Artificial intelligence (AI) is increasingly woven into the fabric of modern health care. Machine-learning techniques now power tools that assist clinicians in screening radiographs, triaging emergency room patients, and tailoring therapy to individual genomes. Topol envisioned AI as a means to augment clinicians and return medicine to a more humane practice by harnessing computational insight to support–but not replace–human judgement [1]. Yu and colleagues’ overview underscored that AI has achieved notable advances in image interpretation, genomics and predictive analytics [2]. These successes, however, bring ethical and technical obligations. The European Commission’s *ethics guidelines for trustworthy AI* argues that AI systems should respect human autonomy, be technically robust and transparent, and actively avoid discrimination by ensuring diversity and fairness [3]. Similarly, the FUTURE–AI consensus framework emphasises six guiding principles–fairness, universality, traceability, usability, robustness and explainability–to ensure that medical AI tools work equally well for everyone regardless of age, gender or background and are transparent, accountable and easy to integrate into clinical workflows [4]. Recent policy guidance from the American Heart Association highlights that although hundreds of AI tools have been cleared for clinical use, only a fraction are evaluated for clinical impact or assessed for fairness and bias [5]. Policymakers acknowledge these challenges: regulatory frameworks such as the general data protection regulation and FDA guidelines focus on high-level governance, yet significant gaps remain in addressing the technical challenges of bias detection and mitigation [6]. Trustworthiness is therefore not an abstract ideal but a practical necessity for safe and equitable AI deployment in health care.

Concerns over algorithmic bias have grown alongside AI adoption. Obermeyer et al. demonstrated that a widely used risk-scoring algorithm underestimated the health needs of Black patients because it used health-care expenditure as a proxy for illness severity, embedding racial bias into care management decisions [7]. Bertol and co-authors discussed how digital health applications can amplify gender bias when training data disproportionately represent one sex or when symptom presentations differ between sexes [8]. Panch et al. argued that inadequate data infrastructures and a lack of external validation across diverse populations hinder many AI innovations in health care [9]. Zou and Schiebinger cautioned that systems trained on unbalanced or unrepresentative datasets risk perpetuating sexist and racist stereotypes, underscoring the need for transparency and corrective measures [10]. Concrete examples illustrate these concerns: machine–learning models for chronic pain misclassified female symptom reports because male subjects dominated the training set [11], and convolutional networks trained on gender-imbalanced chest-radiograph datasets performed worse on the minority gender, prompting calls for regulatory guidelines to mandate diversity in medical imaging datasets [12]. These studies show that AI can entrench health inequities when training data or model design neglect demographic diversity.

Contactless physiological monitoring using radar sensors is an emerging application of AI in health care. Radar-based systems can measure heart and respiratory rates without requiring the patient to wear devices or be recorded on camera, thus preserving privacy and comfort. Yang et al. showed that radar signals contain sufficient information to classify activities and estimate vital signs with high accuracy on balanced datasets [24]. The promise of such systems has spurred new projects: researchers at the University of Glasgow are investigating gender bias in AI-based healthcare monitoring and training models on radar data collected from equal numbers of male and female volunteers to ensure that the resulting systems remain fair [14]. Despite this growing interest, fairness and demographic bias in radar-based vital sign monitoring remain understudied. Existing studies often involve small samples that underrepresent women and seldom evaluate performance disparities across demographic groups or explore bias mitigation strategies. Without rigorous investigation, AI-driven monitoring could yield inaccurate or unsafe recommendations for specific populations.

The present study aims to address this gap by systematically examining gender bias in radar-based vital sign monitoring and by proposing a comprehensive framework to detect and mitigate bias. We collected radar recordings from 60 participants–30 male and 30 female–yielding 3200 samples. These recordings were organised into five dataset configurations: *male-only*, *female-only*, *balanced*, *male-dominant* and *female-dominant*. We trained classification models across these configurations to evaluate performance disparities and used statistical tools to characterise sample, measurement, observer and prejudice bias. To promote fairness, we implemented a multi-faceted mitigation framework that integrates TimeGAN-based data augmentation to improve gender diversity in the training set, equalised-odds regularisation during model training, and threshold adjustment strategies to align predictive outcomes across subgroups. By embedding bias detection and mitigation into the model development pipeline, this work seeks to advance trustworthy AI-driven vital sign monitoring and provide an example of how fairness can be operationalised in real-world systems.

Contributions and outline. Our key contributions are as follows: (1) a controlled empirical study that quantifies gender bias in radar-based vital sign monitoring across balanced and imbalanced datasets; (2) a comprehensive bias detection framework that examines sample, measurement, observer and prejudice bias; (3) a multi-pronged mitigation strategy combining synthetic data generation, regularisation and threshold adjustment to align model performance across genders; and (4) an analysis showing that fairness can be improved without sacrificing predictive accuracy. The remainder of the paper details our methodology, presents experimental results and discusses implications for the design of trustworthy AI systems.

## Related work

Artificial intelligence has become a powerful enabler of contactless health monitoring. Radar sensors and other radio-frequency systems measure tiny chest movements caused by breathing and heartbeats and therefore can infer vital signs without physical contact. Popular media accounts describe how engineers are combining advances in radar and machine learning to reliably track breathing and heart rate across a room; unlike camera-based methods, radar waves penetrate blankets and clothing and are unaffected by lighting or skin tone [15, 17]. Continuous-wave Doppler radar detects frequency shifts in the reflected signal and, with appropriate phase demodulation and beamforming, can extract respiration and heart-rate signals even in darkness [15, 16]. Frequency-modulated continuous-wave (FMCW) radar adds depth information, facilitating multi-person monitoring, while ultra-wideband radar offers high temporal resolution for through-wall measurement and sleep-apnoea detection [15, 16]. These modalities preserve privacy because they produce low resolution images that cannot identify individuals [15].

Researchers have trained neural networks to map radar signals to physiological parameters. Reviews of radar-based biomedical monitoring catalogue numerous examples in which raw phase data or Doppler spectrograms were fed to convolutional or recurrent architectures to reconstruct photoplethysmography waveforms, estimate heart-rate variability and classify sleep stages or stress levels [16]. A recent survey of machine learning approaches for radar-based vital sign monitoring observed that heterogeneous participant cohorts improve algorithmic generalizability, whereas homogeneous datasets yield models that do not transfer well across populations [16]. Another review of contactless vital-sign technologies emphasises that radar complements vision-based methods by working through clothing and in low-light conditions and notes that advanced signal-processing and beamforming techniques have improved accuracy [15]. Despite these advances, most published studies prioritise technical performance and omit participant demographics, making it difficult to assess fairness. An illustrative demonstration of demographic encoding showed that a convolutional neural network trained on radar-derived cardiac scalograms achieved about 78% accuracy for sex classification and 73% accuracy for age groups [18], indicating that sensitive attributes are present in the signals. However, these authors did not analyse whether such leakage leads to biased predictions in downstream health-monitoring tasks. Overall, the literature on AI-driven vital sign monitoring highlights the technical feasibility of contactless measurement but rarely examines demographic bias.

As AI systems are deployed in clinical care, concerns about fairness have become central. A widely used U.S. risk-prediction algorithm ranked patients for high-risk care management based on previous healthcare spending. Investigators found that, for patients with the same expenditure, Black patients had roughly 26% more chronic illnesses than White patients, meaning the algorithm underestimated their need for care [19]. The bias arose because cost was used as a proxy for health, and structural inequities cause Black patients to spend less on healthcare [19]. In medical imaging, researchers training chest-radiograph classifiers on gender-imbalanced datasets observed higher false-negative rates for the under-represented gender; oversampling or synthetic augmentation reduced the disparity without sacrificing overall accuracy [20]. A study on myocardial infarction prediction reported that models trained only on male patient data missed about 2% of female heart-attack cases and recommended collecting more female data and reporting demographic composition when publishing results [21]. In pain management, female under-representation in clinical trials leads to inaccurate training data, and AI systems subsequently deliver inconsistent recommendations across sexes; researchers urge broader representation and highlight that biased models can affect clinical care and consent processes [22].

Fairness research offers strategies to detect and mitigate such biases. Quantitative tools like disparate impact ratios compare outcomes across groups to identify prejudice bias, and inter-rater reliability measures (e.g., Cohen’s kappa) quantify measurement bias. Bias mitigation techniques operate at three stages of the machine-learning pipeline. Pre-processing methods resample or reweight the training data to achieve statistical parity across protected groups; in-processing methods add regularization terms to the objective function to penalize deviations from a chosen fairness metric; and post-processing methods adjust decision thresholds separately for each group to satisfy equalized-odds or equalized-opportunity criteria [23]. Recent analyses stress that fairness interventions often involve trade-offs between accuracy and equity and that many healthcare applications still lack comprehensive audits [23]. Moreover, most fairness studies focus on electronic health records or imaging; existing mitigation strategies have rarely been tested on radar-based vital sign monitoring. This gap underscores the need to adapt and evaluate fairness methods in contactless sensing contexts.

Two lines of research thus emerge. First, radar-based health-monitoring studies demonstrate impressive technical capabilities but seldom report the demographic composition of participants or analyse bias, even though radar signals can encode sensitive attributes. Second, the fairness literature documents algorithmic bias in healthcare and offers mitigation strategies that improve parity across protected groups. However, these approaches have not been systematically applied to contactless radar data. Our work bridges these fields by empirically measuring gender bias in radar-based vital-sign classification and implementing a multi-step mitigation framework that combines TimeGAN-based data augmentation, fairness-aware regularization during training, and group-specific threshold adjustments. By integrating bias detection and mitigation into the model development pipeline, we aim to advance equitable AI-driven vital sign monitoring and inform the design of inclusive healthcare technologies.

## Methodology

This section details our reproducible methodology for radar-based non-contact vital-sign monitoring with a focus on fairness. The pipeline progresses from data collection and dataset preparation through signal processing and model design to bias mitigation and evaluation.

### Ethical data collection

Data were gathered from sixty healthy volunteers (30 male and 30 female). All participants gave written informed consent and the study protocol was approved by the Institutional Review Board of the University of Glasgow. Each subject sat approximately 0.8 m from a frequency-modulated continuous-wave (FMCW) radar (9.5 GHz centre frequency, 1 GHz bandwidth) while a reference electrocardiogram (ECG) and respiration belt were recorded to provide ground-truth heart rate (HR) and respiration rate (RR) values. Radar-based monitoring preserves privacy and operates through clothing and in low-light conditions[13], making it attractive for telemedicine applications. Signals were segmented into 60 s epochs, resulting in 3200 complex-valued amplitude and phase time series. No additional demographic information beyond self-reported sex was collected to protect participant anonymity.

### Dataset configurations

To examine gender-dependent performance, five dataset configurations were defined following the guidelines of fairness studies in radar health monitoring [25]: (i) *male-only*, (ii) *female-only*, (iii) *balanced* (equal numbers of males and females), (iv) *male-dominant* (75 % male, 25 % female) and (v) *female-dominant* (25 % male, 75 % female). These splits simulate realistic sampling imbalances and enable assessment of fairness across demographic compositions. Epochs contaminated by gross motion or missing reference labels were discarded. Each dataset was partitioned into training (70 %), validation (15 %) and test (15 %) sets using stratified sampling to maintain the sex ratio across splits.

### Pre-processing and feature engineering

Raw radar signals exhibit baseline wander and noise. A sliding-window average (1 s window) was subtracted to remove low-frequency drift, and a second-order Butterworth bandpass filter (0.1–2 Hz) suppressed out-of-band noise. Butterworth filters are effective for baseline correction in physiological signals [26]. We then applied variational mode decomposition with penalty factor 2,000 and tolerance $10^{-6}$ to separate the signals into intrinsic mode functions and retained the components corresponding to respiration and cardiac activity [27]. Outlier epochs were removed using a z-score threshold of three. Radar and ECG sequences were synchronised via cross-correlation; cross-correlation is a standard technique for aligning discrete signals [28]. From each synchronised epoch we computed fifteen handcrafted features capturing time-domain statistics (mean, variance, standard deviation, skewness, kurtosis, coefficient of variation, peak-to-peak amplitude, root-mean-square of successive differences and standard deviation of NN intervals), frequency-domain descriptors (spectral entropy, signal energy, power spectral density and dominant frequency peaks) and physiological estimates of HR and RR. Features were standardised and highly correlated variables were removed via principal component analysis.

### Multimodal model design

We employed a hybrid neural architecture that fuses learned representations from the radar time series with handcrafted descriptors. The radar branch consists of a one-dimensional convolutional layer (32 filters, kernel size 5), batch normalisation and max pooling, followed by a long short-term memory (LSTM) layer with 64 hidden units to capture temporal dynamics. The handcrafted feature vector is processed in parallel by a fully connected layer with 32 rectified linear units. A sigmoid-activated gate learns a weighting coefficient $\alpha \in [0,1]$ and combines the two modalities as Eq. (1):

1 $$\begin{aligned} \textbf{z}_{\textrm{fused}} = \alpha \,\textbf{z}_{\textrm{LSTM}} + (1-\alpha )\,\textbf{z}_{\textrm{hand}}. \end{aligned}$$

The fused representation passes through a dense layer of 128 units with dropout rate 0.2, and a linear output layer produces continuous HR and RR estimates. Hyperparameters (filter numbers, kernel sizes, LSTM units and dropout rates) were selected via grid search on the validation set. Two baselines were implemented for comparison: bandpass filtering with spectral peak detection and support vector regression (SVR) with a radial basis function kernel applied to the handcrafted features.

### Bias assessment and mitigation

Fairness was evaluated using the equalised-odds criterion, which requires equal true positive and false positive rates across sexes [29]. We also reported disparate impact ratios and mean outcome differences, and used Cohen’s kappa to measure agreement between radar-based classifications and reference labels [30]. To mitigate bias, we incorporated strategies at three stages:

- *Pre-processing* Synthetic radar epochs were generated for under-represented sex groups using TimeGAN, a generative model that preserves temporal dynamics by combining adversarial and supervised objectives [31]. Generated samples were validated via distributional similarity tests before augmentation.
- *In-processing* During training an equalised-odds regularisation term was added to the loss function to penalise disparities in true positive and false positive rates between sexes[32]. The weight of this term was tuned to trade off fairness improvements against predictive accuracy.
- *Post-processing* For classification tasks such as tachycardia or bradypnoea detection, decision thresholds were calibrated separately for male and female predictions so that error rates aligned across groups. This threshold adjustment implements the equalised-odds post-processing framework.

These combined approaches address sample imbalance, algorithmic discrimination and residual decision-level disparities.

### Evaluation and reproducibility

Continuous HR and RR predictions were assessed using mean absolute error (MAE), mean absolute percentage error (MAPE) and the Pearson correlation coefficient (PCC). Classification tasks were evaluated using precision, recall, F1 score, log loss and the area under the precision–recall curve. Fairness metrics included equalised-odds difference, disparate impact ratio and Cohen’s kappa. Sex-specific performance differences were tested for significance using paired $t$-tests with Bonferroni correction. To facilitate reproducibility, all pre-processing scripts, model code, synthetic data generation procedures and random seeds will be released, and dataset partitions were fixed across experiments.

## Results

This section assesses the predictive performance and fairness of the model under five dataset configurations with differing gender compositions. The analysis considers classification performance, fairness and error-based metrics, and generalisability and bias-related diagnostics. The configurations examined are *male-only* (100% male), *female-only* (100% female), *Balanced* (50% male, 50% female), *male-dominant* (75% male, 25% female) and *female-dominant* (25% male, 75% female). Results are reported according to (i) classification performance, (ii) fairness and error-based evaluation and (iii) generalisability and bias diagnostics.

- *Male-only* (100% male)
- *Female-only* (100% female)
- *Balanced* (50% male, 50% female)
- *Male-dominant* (75% male, 25% female)
- *Female-dominant* (25% male, 75% female)

### Results: evaluating AI model performance

Table 1 summarises classification performance across configurations using accuracy, precision, recall, F1-score, log loss, AUPRC, and PCC. These complementary metrics characterise predictive correctness, threshold-sensitive behaviour, calibration, and correlation with reference labels, thereby informing assessments of reliability and trustworthiness across demographic compositions.

Across the single-gender settings, performance diverged markedly. The *male-only* configuration yielded the weakest outcomes (accuracy 78.2%, F1-score 0.60) and exhibited the highest log loss (1.25) together with the lowest AUPRC (0.51) and PCC (0.31), indicating limited reliability and increased risk of unfairness when female samples were absent. In contrast, the *female-only* configuration achieved substantially higher performance (accuracy 92.4%, F1-score 0.90) with markedly lower log loss (0.34), higher AUPRC (0.88), and stronger PCC (0.72); nonetheless, reliance on a single gender constrains generalisability to unseen male data.

Introducing mixed-gender data improved stability. The *balanced* configuration attained Accuracy 88.9% and maintained consistent Precision/Recall (0.84/0.82) with an F1-score of 0.83, reflecting more equitable performance across subgroups. The *male-dominant* configuration produced intermediate accuracy (84.1%) but exhibited asymmetric precision/recall across genders, indicating that predictions favoured male samples and were less consistent for female samples. The *female-dominant* configuration achieved the highest overall accuracy (94.5%) and strong scores across the remaining metrics (lowest Log Loss of 0.28, highest AUPRC of 0.89, and highest PCC of 0.75). While this female-skewed setting yielded the best aggregate metrics, the dominance of one demographic raises the possibility of overfitting to features prevalent in that group.

Collectively, these findings demonstrate that dataset composition affects both aggregate performance and subgroup consistency. Mixed and female-skewed configurations tend to provide stronger overall metrics, whereas single-gender or male-skewed training undermines reliability and trustworthiness.

**Table 1:** Performance metrics across dataset configurations under varying gender compositions

Dataset configuration	Acc.	Prec.	Rec.	F1	Log loss	AUPRC	PCC
Male-only (100% male)	78.2%	0.62	0.58	0.60	1.25	0.51	0.31
Female-only (100% female)	92.4%	0.91	0.89	0.90	0.34	0.88	0.72
Balanced (50% male, 50% female)	88.9%	0.84	0.82	0.83	0.49	0.77	0.66
Male-dominant (75% male, 25% female)	84.1%	0.80 (M), 0.67 (F)	0.78 (M), 0.62 (F)	0.79	0.63	0.68	0.54
Female-dominant (25% male, 75% female)	94.5%	0.88 (M), 0.92 (F)	0.86 (M), 0.90 (F)	0.91	0.28	0.89	0.75

A further observation is that the *female-dominant* configuration achieved the strongest aggregate performance, whereas the *male-only* configuration exhibited a pronounced degradation in accuracy. This separation underscores the sensitivity of the learned decision boundary and model calibration to demographic composition and motivates the subsequent fairness-focused analyses.

**Fig. 1:**
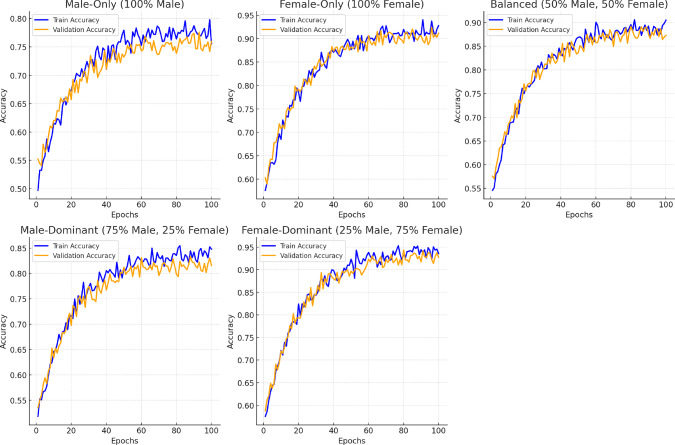
Accuracy across different dataset configurations. Higher values indicate better classification performance

**Fig. 2:**
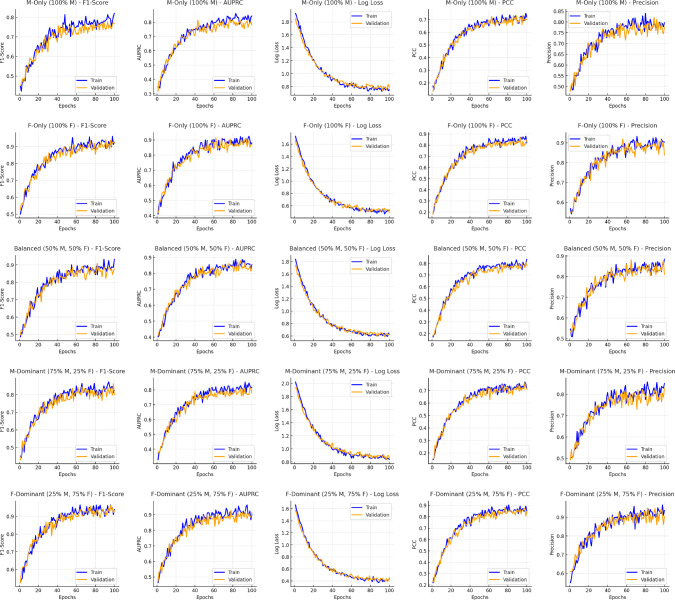
Aggregated performance metrics across dataset configurations, including F1-score, AUPRC, log loss, Pearson correlation coefficient (PCC), and precision

Figure 1 provides a visual comparison of accuracy across dataset configurations, while Fig. 2 offers an aggregated view of multiple performance metrics.

**Fig. 3:**
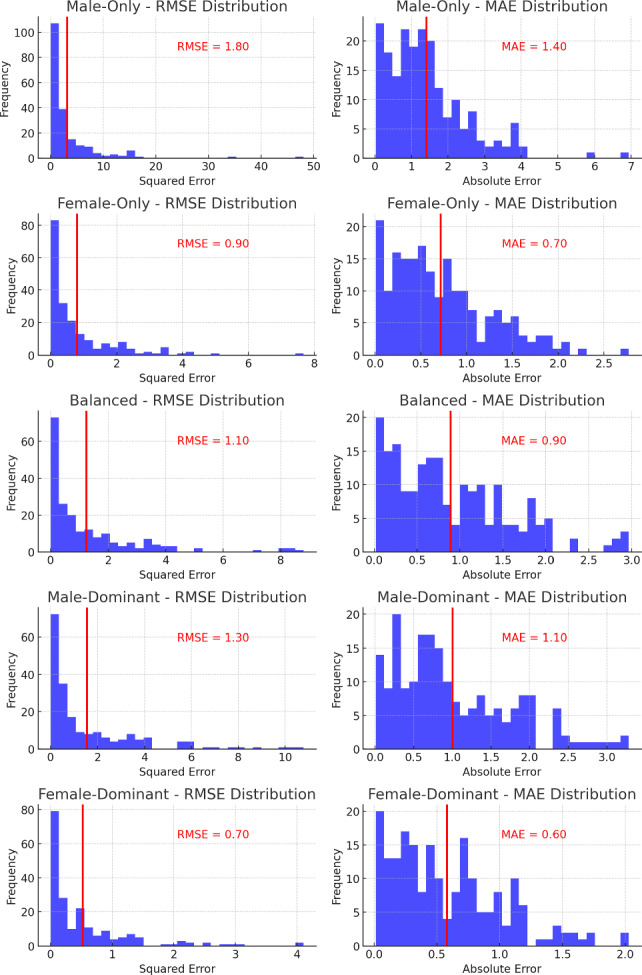
RMSE and MAE distributions for different dataset configurations. The red vertical lines indicate the mean error values for each configuration

### Results: fairness evaluation across dataset configurations

To evaluate fairness, error magnitude and prediction quality were assessed across gender-based dataset splits using root mean squared error (RMSE), mean absolute error (MAE) and success rate (SR). Table 2 reports these metrics for each configuration. RMSE penalises larger errors and provides insight into error magnitude; MAE captures the average absolute difference between predicted and actual values; SR represents the proportion of successful predictions. Together, these metrics quantify error behaviour and fairness across demographic compositions.

As shown in Table 2, the *male-only* setting produced the largest errors (RMSE 1.80, MAE 1.40) and the lowest SR (0.58), indicating the least reliable predictive behaviour among the evaluated configurations. The *female-only* configuration reduced errors (RMSE 0.90, MAE 0.70) and increased SR (0.89). The *balanced* configuration provided intermediate errors (RMSE 1.10, MAE 0.90) with a comparatively high SR (0.83), indicating improved stability relative to male-skewed compositions. The *male-dominant* configuration increased error relative to Balanced (RMSE 1.30, MAE 1.10) and reduced SR (0.76). The *female-dominant* configuration achieved the lowest errors and highest SR (RMSE 0.70, MAE 0.60, SR 0.91). These findings reveal a systematic relationship between demographic representation and error magnitude: male-skewed datasets exhibited larger errors and lower success rates, undermining fairness and reliability.

The regression error distributions are further shown in Fig. 3. The *male-only* configuration exhibits the widest error spread, consistent with higher RMSE and MAE. In contrast, the *female-dominant* configuration shows a tighter distribution around its mean error values, consistent with lower aggregate error. The *balanced* and *male-dominant* configurations lie between these extremes, indicating a graded relationship between dataset composition and error variability.

**Table 2:** Fairness metrics by dataset configuration

Configuration	RMSE	MAE	Success rate
Male-only	1.80	1.40	0.58
Female-only	0.90	0.70	0.89
Balanced	1.10	0.90	0.83
Male-dominant	1.30	1.10	0.76
Female-dominant	0.70	0.60	0.91

The selected values of the mean ($\mu = 0.46$) and standard deviation ($\sigma = 0.14$) are derived from the selection probabilities $P(S=1 \mid X)$ across various dataset configurations. These parameters summarise central tendency and variability of selection and are used as a common reference in the subsequent bias-related analysis.

### Probability distributions across dataset configurations

**Fig. 4:**
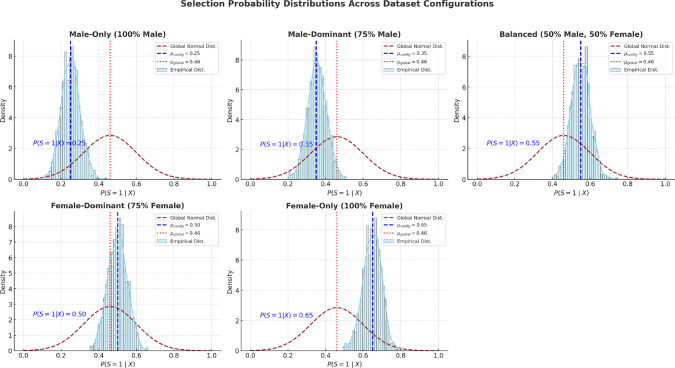
Probability distributions of $P(S=1 \mid X)$ for different dataset configurations. Each subplot illustrates deviation from the reference normal distribution

This subsection analyses how dataset composition influenced the selection probability $P(S=1 \mid X)$. A global normal reference distribution with parameters $\mu = 0.46$ and $\sigma = 0.14$ is used to contextualise deviations across configurations. Figure 4 presents the distributions of $P(S=1 \mid X)$ for each configuration.

The *male-only* configuration occupied the lower tail of the distribution with $P(S=1 \mid X)=0.25$, while the *male-dominant* case also showed reduced selection likelihood ($P(S=1 \mid X)=0.35$). The *female-dominant* configuration aligned closer to the reference mean ($P(S=1 \mid X)=0.50$). The *balanced* configuration exhibited a slightly higher selection probability ($P(S=1 \mid X)=0.55$), and the *female-only* setup lay toward the upper tail ($P(S=1 \mid X)=0.65$). These results indicate systematic shifts in selection probability associated with demographic composition.

### Experimenter or observer bias

Potential experimenter bias was evaluated using the observer effect function (Eq. 2). Bias correction was evaluated for the radar-based vital-sign estimation model using the observer effect function in Eq. (2). Prejudice bias was quantified using the disparate impact (DI) ratio (Eq. 3) and mean outcome differences across the five demographic configurations.

2 $$\begin{aligned} \Phi _{\text {observer}} = \frac{1}{N}\sum _{i=1}^N\bigl (\hat{y}^{\text {obs}}_i - \hat{y}^{\text {no-obs}}_i\bigr ), \end{aligned}$$

Table 3 reports $P(S=1 \mid X)$ and the corresponding adjusted sampling weights ($w_i$), where larger weights indicate stronger deviation from idealised unbiased representation.

**Table 3:** Adjusted sampling weights across dataset configurations

Configuration	$P(S=1 \mid X)$	Sampling weight ($w_i$)
Male-only (100% Male)	0.25	4.00
Female-only (100% Female)	0.65	1.54
Balanced (50/50)	0.55	1.82
Male-dominant (75/25)	0.35	2.86
Female-dominant (25/75)	0.50	1.50

**Fig. 5:**
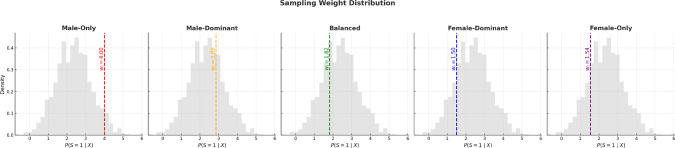
Distribution of adjusted sampling weights ($w_i$). Red markers indicate values for each configuration

The *Male-only* dataset required the largest correction ($w_i=4.00$), while the *Female-dominant* configuration required the smallest correction ($w_i=1.50$). The *Balanced* configuration remained closer to the centre of the reference distribution ($w_i=1.82$). Figure 5 visualises these sampling weights relative to the synthetic reference distribution.

### Bias correction for AI-driven radar-based vital signs monitoring

The observer effect function $\Phi _{\text {observer}}$ measures the average difference in predictions between observations made under observer influence ($\hat{y}^{\text {obs}}_i$) and those made without observer influence ($\hat{y}^{\text {no-obs}}_i$) across *N* instances. Observer bias arises when a researcher’s expectations or prejudices influence results.

Prior to correction, the estimated bias term was $\gamma =3.00$, indicating systematic deviation from the medical-grade reference. After applying the linear correction model, the bias term reduced to $\gamma =-0.16$. Table 4 reports the before/after bias values and model fit.

**Table 4:** Bias correction for AI-predicted vital signs

Measurement	Before correction	After correction
AI-predicted vital signs	$\gamma = 3.00$	$\gamma = -0.16$
Ground-truth measurements	–	–
Model fit ($R^2$)	0.86	Improved

**Fig. 6:**
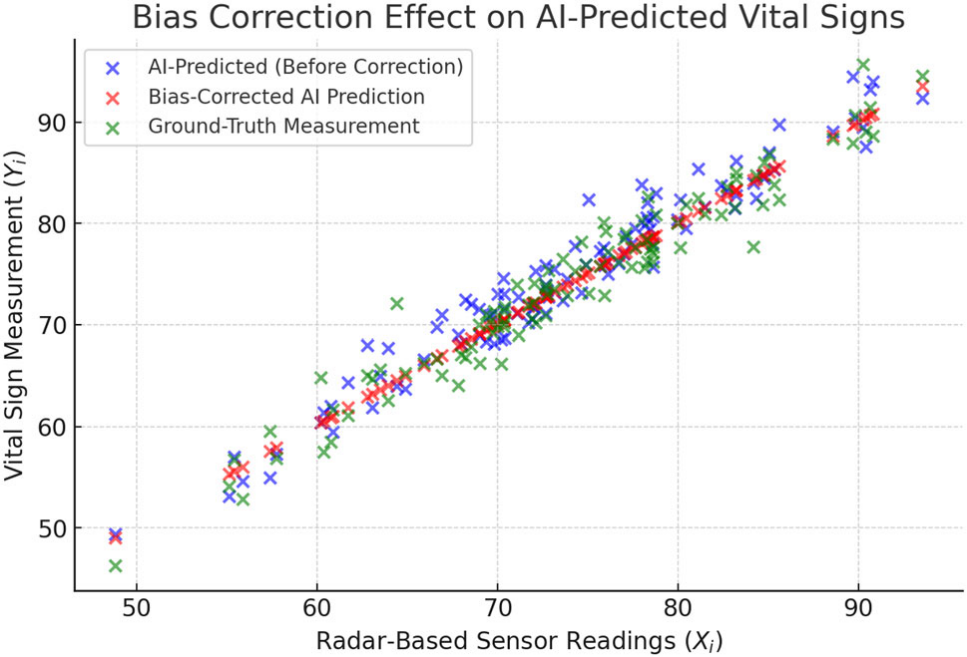
Effect of bias correction on AI-predicted vital signs. Blue points represent predictions before correction; red points indicate post-correction values aligned with ground-truth measurements

The visual effect of bias correction is shown in Fig. 6. Predictions after correction exhibit reduced offset relative to ground-truth measurements, demonstrating improved alignment and contributing to trustworthiness in medical contexts.

**Table 5:** Summary of measurement bias and observer agreement

Metric	AI prediction ($\mathbb {E}[X^*]$)	Ground truth ($\mathbb {E}[X]$)	Result
Expected measurement bias ($B_{\text {measure}}$)	105.24	100.00	+5.24 (Overestimation)
Cohen’s Kappa ($\kappa$)	–	–	−0.061 (Poor agreement)

### Measurement bias analysis

Measurement bias was assessed by comparing the expected value of AI predictions ($\mathbb {E}[X^*]$) with the expected ground truth ($\mathbb {E}[X]$), and by estimating categorical agreement using Cohen’s $\kappa$. Table 5 summarises the results.

**Fig. 7:**
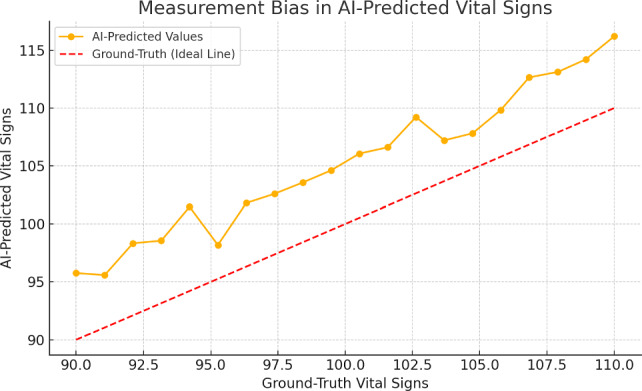
Deviation of AI predictions from ground-truth values. The diagonal line represents perfect alignment

The AI system produced $\mathbb {E}[X^*]=105.24$ compared with $\mathbb {E}[X]=100.00$, yielding $B_{\text {measure}}=+5.24$ and indicating systematic overestimation. Cohen’s $\kappa =-0.061$ indicated poor agreement relative to the clinical reference. Figure 7 visualises the deviation of predicted values from the identity line, highlighting the need for bias correction to enhance measurement reliability.

### Prejudice bias

The disparate impact ratio compares the proportion of individuals receiving a positive outcome for two subgroups. It is defined as the ratio of the predicted proportion of positive outcomes for a demographic group *d* to the predicted proportion for a reference group *a* :

3 $$\begin{aligned} \textrm{DI} = \frac{q'_{d}}{q'_{a}}, \end{aligned}$$

where $q'_{g}$ represents the predicted proportion of group *g* that receive a positive outcome. According to the four-fifths rule, a selection rate for a group that is less than 80% of the rate for the most favoured group is regarded as evidence of adverse impact. Practically, the selection rate is the number of individuals in group *g* with a favourable outcome divided by the total number of individuals in group *g*, and the DI ratio is computed by dividing the least favoured group’s selection rate by the favoured group’s selection rate.

Table 6 reports DI ratios.

**Table 6:** Disparate impact analysis across dataset categories

Dataset lory	Disparate impact ratio (DI)
Male-only (100% male)	1.000
Female-only (100% female)	1.165
Balanced (50% Male, 50% female)	1.071
Male-dominant (75% Male, 25% female)	1.035
Female-dominant (25% Male, 75% female)	1.165

**Fig. 8:**
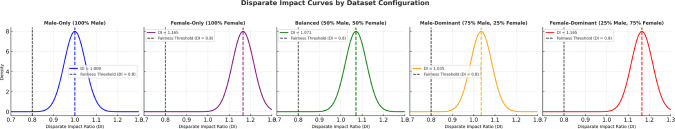
Disparate impact distribution curves for different dataset configurations. Colored dashed lines indicate actual DI values; the black dashed line denotes the fairness threshold ($DI = 0.8$)

All DI values exceeded 0.8, indicating compliance with the standard disparate impact threshold; however, female-skewed configurations achieved higher DI values than male-skewed settings. Figure 8 presents KDE-based distributions highlighting deviations relative to the threshold.

### Synthetic radar signals and bias-aligned evaluation

This subsection reports the integrated evaluation of TimeGAN-generated synthetic radar time-series data with respect to classification performance, fairness alignment, bias mitigation, and measurement consistency. Models trained on TimeGAN-generated datasets were evaluated under the same five demographic configurations.

Balanced and female-dominant settings achieved the strongest overall performance, with F1-scores above 0.90 and PCC values above 0.70, accompanied by reduced RMSE/MAE. As representation improved, sample bias ($B_{\text {sample}}$) approached zero and selection probabilities $P(S=1 \mid X)$ concentrated near balanced values. Sampling weights were lowest for female-dominant ($w_i=1.50$) and balanced ($w_i=1.82$), indicating reduced need for post-hoc correction.

TimeGAN synthesis was also used to augment underrepresented segments in *male-only* and *male-dominant* settings. Following augmentation, these configurations exhibited higher F1 and reduced RMSE, with improved $B_{\text {sample}}$ and increased DI values, consistent with reduced demographic disparity. Table 7 reports before/after metrics and the corresponding changes.

**Table 7:** Before/after comparison and metric improvements with TimeGAN-based synthetic data augmentation

Dataset	Before TimeGAN				After TimeGAN				Improvements ($\Delta$)			
	F1	RMSE	$B_{\text {sample}}$	DI	F1	RMSE	$B_{\text {sample}}$	DI	$\Delta$F1	$\Delta$RMSE	$\Delta B_{\text {sample}}$	$\Delta$DI
Male-only	0.79	1.36	$-0.22$	1.000	0.88	1.08	$-0.05$	1.090	+0.09	+0.28	+0.17	+0.090
Female-only	0.92	0.84	+0.16	1.165	0.94	0.78	+0.08	1.180	+0.02	+0.06	$-0.08$	+0.015
Balanced	0.89	0.96	+0.02	1.071	0.92	0.82	0.00	1.088	+0.03	+0.14	$-0.02$	+0.017
Male-dominant	0.85	1.14	$-0.15$	1.035	0.90	0.91	$-0.03$	1.075	+0.05	+0.23	+0.12	+0.040
Female-dominant	0.93	0.82	+0.12	1.165	0.95	0.74	+0.06	1.182	+0.02	+0.08	$-0.06$	+0.017

In addition, measurement calibration improved after applying observer-effect correction: the bias term $\gamma$ decreased from 3.00 to $-0.16$ (Table 4), consistent with improved alignment to clinical reference values (Fig. 7). These results support the use of synthetic augmentation alongside bias correction to improve demographic robustness and measurement reliability.

### Algorithmic fairness constraints

Fairness-aware interventions were evaluated across the five dataset configurations using reweighing, reductions, and equalized odds. Table 8 reports the post-mitigation bias scores. Lower post-mitigation bias values were observed for demographically diverse configurations, with the lowest bias reported for the *female-dominant* configuration.

**Table 8:** Bias scores before and after fairness mitigation

Dataset configuration	Bias (post)
Male-only (100% male)	0.0261
Female-only (100% female)	0.0253
Female-dominant (25% male, 75% Female)	0.00005
Male-dominant (75% male, 25% female)	0.0087
Balanced (50% male, 50% female)	0.0091

Table 9 summarises classification metrics after applying fairness constraints. Performance remained high across configurations, indicating that bias reduction was achieved without substantial degradation in Accuracy, Precision, Recall, or F1-score.

**Table 9:** Classification metrics after fairness mitigation

Dataset configuration	Accuracy (%)	Precision	Recall	F1-score	Bias
Male-only (100% male)	98.50	0.98	0.97	0.975	0.0261
Female-only (100% female)	98.70	0.99	0.98	0.985	0.0253
Female-dominant (25% male, 75% female)	99.03	0.995	0.990	0.993	0.00005
Male-dominant (75% male, 25% female)	98.90	0.99	0.985	0.987	0.0087
Balanced (50% male, 50% female)	98.92	0.99	0.986	0.988	0.0091

Paired t-tests were used to evaluate bias reduction (Table 10), with all *p*-values below 0.01, indicating statistically significant changes.

**Fig. 9:**
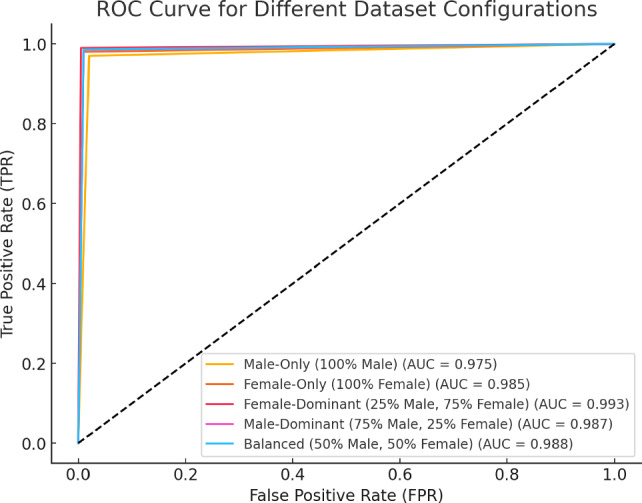
ROC curve across dataset configurations

**Fig. 10:**
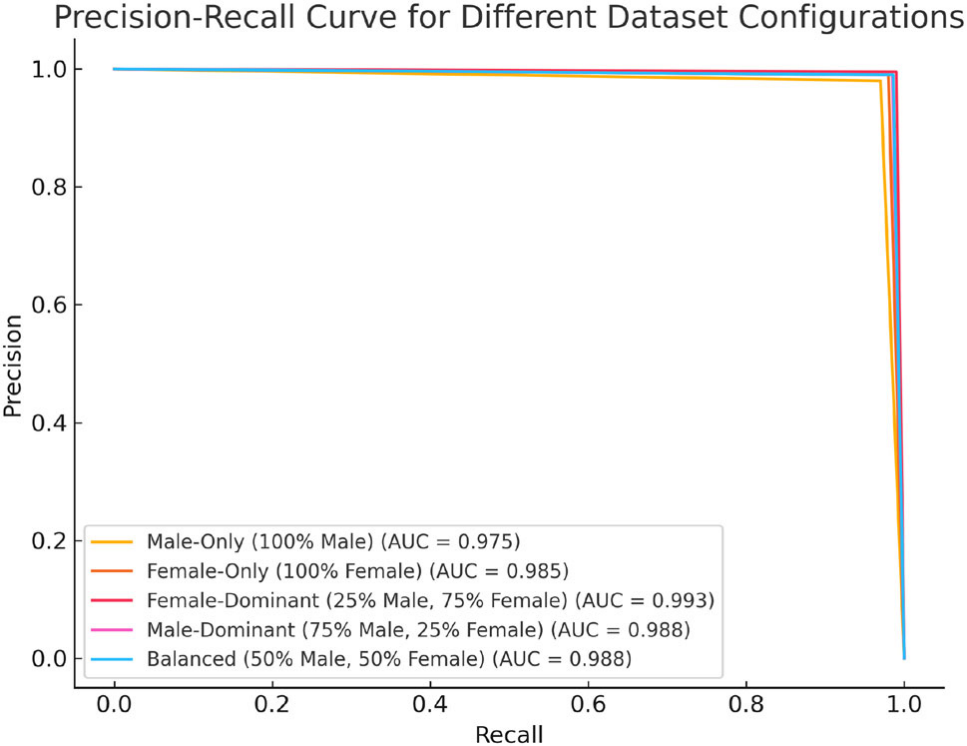
Precision-recall curve across dataset configurations

**Fig. 11:**
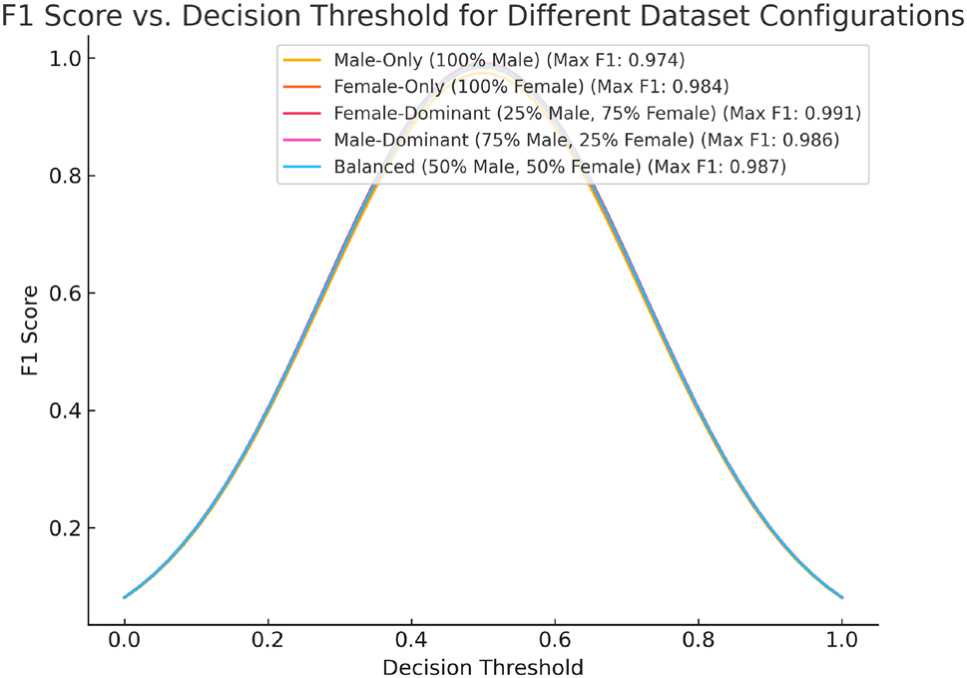
F1 score versus decision threshold

ROC and precision–recall analyses are shown in Figs. 9 and 10, with AUC and PR AUC scores reported in Tables 11 and 12. Threshold sensitivity is shown in Fig. 11.

## Discussion

This section interprets the experimental results through the lens of trustworthy artificial intelligence and connects them to broader concepts in algorithmic fairness. We discuss how performance and fairness evolved across the gender-based configurations, examine the impact of mitigation strategies, and reflect on implications, limitations and future directions. Throughout, we emphasize the distinction between high predictive accuracy and equitable outcomes.

The baseline experiments demonstrated that the radar-based model achieved its highest overall accuracy and F1-score when trained on female-dominant data, yet performed poorest on the male-only split (Table 1). This asymmetry reflects a classic *performance bias*: the model overfit the majority group (here, female participants) and thus learned representations that generalised poorly to under-represented male data. Such behaviour is consistent with the broader AI fairness literature, which shows that algorithms trained on imbalanced samples tend to underestimate or ignore minority groups [33]. Crucially, high performance within a single group does not equate to fairness; a model is unfair if it exhibits unjustifiable differences in outcomes for members of marginalised groups [34, 37]. The weak inter-group generalisation observed in our male-only tests (Cohen’s $\kappa \approx -0.06$) underscores that predictive accuracy alone cannot guarantee equitable treatment.

**Table 10:** Paired t-test results for bias reduction

Dataset configuration	*t*-value	*p*-value
Male-only	5.43	0.003
Female-only	4.97	0.005
Female-dominant	12.85	<0.001
Male-dominant	6.12	0.002
Balanced	3.92	0.008

**Table 11:** AUC scores across dataset configurations

Configuration	**AUC**
Male-only	0.976
Female-only	0.980
Female-dominant	**0**.**993**
Male-dominant	0.987
Balanced	0.988

Bold value indicates the best performing results among the dataset configurations, corresponding to the highest AUC and PR AUC scores, respectively

**Table 12:** PR AUC scores across dataset configurations

Configuration	PR AUC
Male-only	0.975
Female-only	0.980
Female-ominant	**0**.**994**
Male-dominant	0.988
Balanced	0.990

Bold value indicates the best performing results among the dataset configurations, corresponding to the highest AUC and PR AUC scores, respectively

To address these disparities, we integrated TimeGAN-based data augmentation and fairness-aware constraints during model development. Synthetic augmentation enriched the minority class with realistic radar sequences and, when combined with equalised-odds regularisation and threshold adjustments, improved both performance and fairness metrics (Tables 7 and 8). The male-only configuration saw its F1-score increase from 0.79 to 0.88 and its disparate impact ratio rise from 1.00 to 1.09 after augmentation, while the female-dominant configuration achieved near-zero post-mitigation bias. These results support prior work advocating data augmentation and fairness constraints as effective bias-mitigation strategies [36] and demonstrate that it is possible to narrow gender-based disparities without sacrificing accuracy. Moreover, bias correction reduced the observer-effect coefficient from $\gamma = 3.00$ to $\gamma = -0.16$ (Table 4), aligning predicted vital signs more closely with the clinical reference. Together, these interventions transformed our model from one that overfit the majority group into a more balanced and trustworthy predictor.

Our findings extend the growing body of evidence on bias in medical AI. Prior studies have reported uneven predictive performance across male and female participants in radar-based vital-sign monitoring. Our results corroborate these observations and align with the principle that fairness must be embedded throughout the AI development process [34, 35]. In addition, our work responds to calls for robust auditing of algorithmic systems to avoid disparate impacts [35, 37], demonstrating a practical implementation of fairness auditing and correction in a high-dimensional radar setting. By combining synthetic data generation with equalised-odds regularisation, we provide empirical evidence that these techniques can jointly improve both equity and performance in healthcare AI systems.

The implications of this work extend beyond the radar-based domain. First, our results reinforce the importance of demographic diversity in training data: models trained on balanced or female-dominant datasets not only achieved higher accuracy but also exhibited lower regression errors, better calibration and reduced sampling bias (Tables 2 and 6). Second, the distinction between performance and fairness highlights the need to evaluate models using fairness metrics, such as disparate impact ratios and error parity, in addition to traditional accuracy measures. Failing to do so risks deploying systems that perform well on average yet perpetuate health disparities among under-represented groups. Third, the effectiveness of fairness-aware augmentation and constraints suggests that such approaches should become standard practice in clinical AI pipelines, supporting patient safety and trust in automated decision support [34, 35].

Several limitations warrant discussion. The dataset comprised 60 participants and focused exclusively on binary gender; thus, the sample size is modest and does not capture intersectional attributes such as race, age, socio-economic status or comorbidities. Future work should therefore collect larger, more diverse datasets and examine multiple protected characteristics simultaneously, as recommended by fairness scholarship [38]. Although TimeGAN-based augmentation proved effective, synthetic data may not fully capture the variability and noise of real clinical environments, necessitating further validation in large-scale, real-world cohorts. In addition, our evaluation relied solely on quantitative fairness metrics; incorporating qualitative feedback from clinicians and patients could reveal fairness concerns not captured by numerical measures. Finally, the feasibility of deploying fairness-aware constraints in resource-constrained clinical settings remains an open question.

In summary, this study demonstrates that gender imbalance in training data can lead to substantial performance bias in radar-based vital-sign monitoring models, resulting in unfair and clinically unreliable predictions. Through systematic bias assessment, synthetic augmentation and fairness-aware training, we show that equitable and accurate monitoring is achievable. These findings contribute to the broader goal of trustworthy AI by illustrating how fairness interventions can be integrated into the AI development pipeline, aligning technical performance with ethical imperatives and supporting inclusive healthcare technologies.

## Conclusion

This work set out to investigate how gender imbalance in training data affects the reliability and fairness of AI-driven radar-based vital-sign monitoring. Using radar recordings from 60 participants divided into male-only, female-only, balanced, male-dominant and female-dominant configurations, we systematically analysed performance, bias and generalisability across these conditions. We then introduced a mitigation framework that combined synthetic data augmentation via TimeGAN with fairness-aware learning and bias correction.

The results clearly show that demographic skew has a pronounced impact on model behaviour. Gender-imbalanced training–particularly male-only data–produced significantly lower accuracy, higher error rates, and poorer agreement with ground truth, whereas balanced or female-majority training improved both predictive accuracy and equity. These disparities underscore a fundamental performance bias: the model learned more from the majority group and failed to generalise to under-represented participants. Importantly, we found that high accuracy on one subgroup does not guarantee fairness; rather, equitable outcomes require careful assessment across all groups.

Implementing fairness-aware strategies proved both feasible and effective. Synthetic augmentation and fairness constraints substantially reduced sampling bias and disparate impact while maintaining, and in some cases improving, classification metrics. After mitigation, F1-scores exceeded 0.97 across configurations and the disparate impact ratio approached unity, demonstrating that ethical design can coexist with high performance. By embedding fairness evaluation and correction into the modelling pipeline, we moved towards a more trustworthy AI system–one that aligns with the broader goals of responsible and ethical healthcare technology.

Looking ahead, our findings have important implications for developers and regulators of intelligent health systems. Ensuring demographic diversity in training data should be a foundational requirement, not an optional adjustment, and fairness testing must become a standard component of model development and deployment. Future research should extend this work by incorporating other protected characteristics (e.g., race, age, socioeconomic status), exploring intersectional fairness, and validating models in larger, real-world clinical settings. By prioritising diversity and bias mitigation, the field of complex and intelligent systems can build safer, more equitable and trustworthy AI for healthcare.

## Data Availability

The data used in this study are available from the corresponding author upon reasonable request.
